# Integrating heterogeneous drug sensitivity data from cancer pharmacogenomic studies

**DOI:** 10.18632/oncotarget.10010

**Published:** 2016-06-14

**Authors:** Nikita Pozdeyev, Minjae Yoo, Ryan Mackie, Rebecca E. Schweppe, Aik Choon Tan, Bryan R. Haugen

**Affiliations:** ^1^ Department of Medicine, University of Colorado Cancer Center, University of Colorado School of Medicine, Aurora, CO, USA

**Keywords:** database integration, drug sensitivity, pharmacogenomics, cancer, cell line

## Abstract

The consistency of *in vitro* drug sensitivity data is of key importance for cancer pharmacogenomics. Previous attempts to correlate drug sensitivities from the large pharmacogenomics databases, such as the Cancer Cell Line Encyclopedia (CCLE) and the Genomics of Drug Sensitivity in Cancer (GDSC), have produced discordant results. We developed a new drug sensitivity metric, the area under the dose response curve adjusted for the range of tested drug concentrations, which allows integration of heterogeneous drug sensitivity data from the CCLE, the GDSC, and the Cancer Therapeutics Response Portal (CTRP). We show that there is moderate to good agreement of drug sensitivity data for many targeted therapies, particularly kinase inhibitors. The results of this largest cancer cell line drug sensitivity data analysis to date are accessible through the online portal, which serves as a platform for high power pharmacogenomics analysis.

## INTRODUCTION

Systematic pharmacogenomics studies of large panels of cancer cell lines have proven to be a useful pre-clinical resource in identifying clinically relevant drugs and putative predictive biomarkers in cancer research. For example, the Cancer Cell Line Encyclopedia (CCLE) [1], the Genomics of Drug Sensitivity in Cancer (GDSC) [2] and the Cancer Therapeutics Response Portal (CTRP) [3] projects have systematically collected drug sensitivity profiles and genomic information across hundreds of compounds and cancer cell lines. These studies have recapitulated known drugs and cancer-dependency relationships as well as revealed novel biomarkers associated with the drug sensitivity [1–4]. As large-scale pharmacogenomics studies are becoming more available, integrating these various data sets will provide a rich resource for the robust novel hypothesis generation and discovery in cancer therapeutics development. However, integrating these diverse pharmacogenomics studies poses a methodological and analytical challenge due to the differences in the types of assays, maximum tested drug concentration, the range of tested drug concentrations and drug sensitivity metrics employed by different studies. These challenges were highlighted, when Haibe-Kains et al. found inconsistencies in drug sensitivity data from CCLE and GDSC [5]. In response, the authors of CCLE and GDSC re-analyzed drug sensitivity data accounting for the differences in the analytical approach employed by the two studies and reported a significantly better agreement [6].

In this study, we developed a new metric, area under the dose-response curve adjusted for the range of tested concentrations (adjusted AUC), to combine heterogeneous drug sensitivity data generated by multiple pharmacogenomics studies. To illustrate the utility of this new metric, we performed an unbiased comparison of the drug screening data from CCLE, GDSC and CTRP, resolved analytical challenges of the previous comparative analyses, and defined the variables associated with the better or worse agreement of the drug sensitivities between studies. We also developed a companion online portal, the Quantitative Analysis of Pharmacogenomics in Cancer (QAPC, http://tanlab.ucdenver.edu/QAPC), to explore and download the summarized data for high-power pharmacogenomic analysis by the scientific community.

## RESULTS AND DISCUSSION

**Figure 1:**
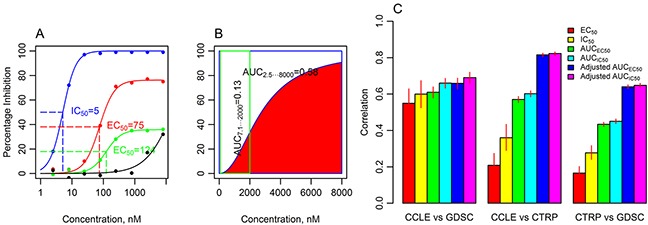
The six drug sensitivity metrics and the agreement of pooled pharmacologic data between CCLE, GDSC and CTRP databases **A.** An IC_50_ dose-response curve has minimal (*A_min_*) and maximal (*A_max_*) asymptotes set to 0 and 100, respectively (blue line, CCLE, paclitaxel, cell line REH); EC_50_ is estimated from the dose response data with flexible *A_min_* and *A_max_* (red line, CCLE, selumetinib, cell line A375), but EC_50_ is ambiguous for low amplitude curves (green line, CCLE, selumetinib, cell line 639V, see detailed explanation in the text). The IC_50_ or EC_50_ cannot be estimated from incomplete dose response curve (black line, CCLE, selumetinib, cell line EFO27). **B.** AUCs calculated from the dose response curve (CCLE, crizotinib, cell line KMS26) using the range of drug concentrations from CCLE (2.5-8000 nM, blue box) and GDSC (7.8125-2000 nM, green box) differ (0.58 and 0.13, respectively). Adjusted AUC is calculated for the range of concentrations shared by the databases (green box). **C.** Pearson correlation coefficients (*r*) for the comparison of drug sensitivity data in 3 databases using six drug sensitivity metrics. Pearson correlation was calculated for a subset of EC_50_ and IC_50_ with finite values (estimated to be within the range of tested concentrations). Adjusted AUC provides the best consistency among databases. Error bars illustrate 95% confidence intervals (random permutations).

The CCLE and GDSC projects used half maximal inhibitory concentration (IC_50_), defined as a drug concentration producing absolute 50% inhibition of growth in the proliferation assay (Figure 1A, blue). By definition, this metric relies on the assumption, that at a high concentration of the drug, 100% effect is achieved (all cells die in a proliferation assay). This is true for potent cytotoxic drugs such as doxorubicin and paclitaxel (see examples in the QAPC portal, http://tanlab.ucdenver.edu/QAPC). However, cancer pharmacogenomics increasingly focuses on the targeted therapies, and many of these drugs, such as MEK inhibitors, are cytostatic [7]. Dose response curves produced for cytostatic drugs, such as MEK inhibitor selumetinib, plateau at a percent inhibition (maximal drug effect, A_max_) of less than 100 % (Figure 1A, red). 4-point logistic regression can estimate lower and upper asymptotes of the sigmoid dose-response curve that differ from 0 and 100 % of an IC_50_ curve and calculate half maximal effective concentration (EC_50_, also called relative IC_50_), which is defined as a concentration of a drug causing an effect equal to the 50% of the A_max_. More flexible, EC_50_ curves model experimental data better (median residuals standard error is 7.0 and 9.2 for EC_50_ and IC_50_ curve fits, respectively, Mann-Whitney-Wilcoxon, p< 0.001, n = 478086) and may provide more accurate measure of the drug sensitivity for cytostatic drugs. However, EC_50_ is ambiguous for the low amplitude curves (Figure 1A, green). Such a curve may represent a true low amplitude drug response to a cytostatic drug, a beginning part of the high amplitude dose response curve with an EC_50_ exceeding maximal tested concentration, or a signal drift due to the technical imperfections of the large scale screening.

Incomplete dose response curves (Figure 1A, black) present a major challenge in interpreting drug sensitivity data. Neither IC_50_ nor EC_50_ can be accurately estimated from these drug responses. Other proposed drug sensitivity metrics, such as Hill slope and A_max_ [8] cannot be used as well. The three pharmacogenomic projects handled this problem differently. The CCLE assigned an IC_50_ of 8 μM (maximal tested concentration) to incomplete dose response curves. This approach provides a finite number for the downstream analysis, but capped IC_50_ values are not always supported by the experimental data. The GDSC extrapolated data from incomplete curves outside of the range of tested concentrations that produced high and inaccurate IC_50_ values. The CTRP used AUC as a measure of drug sensitivity, but not IC_50_ or EC_50_.

Haibe-Kains et al. compared CCLE and GDSC IC_50_ data as published without considering the differences in methodology [5]. This resulted in the expected poor agreement between the two datasets. To account for the analytical differences, Stransky et al. capped IC_50_ at a maximal drug concentration tested by the GDSC study (Supplementary Table S1) [6]. This produced a large number of capped IC_50_ values, which are not supported by the experimental data for the 15 drugs shared by the two databases (Supplementary Table S2), and overestimated the correlation between IC_50_ values. For the drugs reported to have the greatest IC_50_ correlation between CCLE and GDSC, such as crtizotinib, lapatinib, and nilotinib (Pearson correlation *r* = 0.74, 0.78, 0.89, respectively), up to 98% of IC_50_ values were capped (Supplementary Table S2)). Since the GDSC used lower maximal tested concentrations for 14 out of 15 drugs shared with CCLE (Supplementary Table S1), a portion of reliable CCLE IC_50_ estimates (IC_50_ values within the range of tested drug concentrations) were capped, eliminating potentially useful drug sensitivity information. For the drugs with the least number of capped IC_50_ values (17-AAG and paclitaxel, 19 % and 29 % capped IC_50_ values, respectively), no improvement in the correlation was found by Stransky et al. (0.57 and 0.15 for 17-AAG and paclitaxel, respectively) when compared to Haibe-Kains et al. analysis (0.61 and 0.16 for 17-AAG and paclitaxel, respectively). In addition, four drugs analyzed by both studies have more than one maximal tested concentration in the GDSC dataset, adding ambiguity to this artificial IC_50_ cap (Supplementary Table S1). The above considerations suggest that IC_50_ calculations (and EC_50_ calculations, following the same logic) are not suitable drug sensitivity metrics to accurately compare and reconcile drug sensitivities from the large pharmacogenomic studies, which contain many incomplete dose response curves.

The AUC, on the other hand, (Figure 1B, red area) is an attractive drug sensitivity metric, as it can be calculated for any dose response curve. AUC is unambiguous, combines information about the potency (EC_50,_ IC_50_) and efficacy (A_max_) of the drug into a single measure, and has been shown to be a robust metric for comparing a single drug across cell lines [8], as well as a better measure of cell line selectivity, when compared to IC_50_ [9]. However, AUC depends on the range of tested drug concentrations, which varies between studies. Figure 1B illustrates that AUC calculated from the same dose-response data for the range of concentrations used by GDSC (7.8-2000 nM) and CCLE (2.5-8000 nM) differs by more than 4-fold (0.13 and 0.58 respectively). The influence of the range of concentration on AUC estimate is particularly obvious, when maximal tested concentration differs significantly between databases, such as for crizotinib. For example, the median AUC calculated from the IC_50_ model of crizotinib reponses in our analysis were 0.02, 0.14 and 0.84 for GDSC, CCLE and CTRP, respectively, reflecting the marked differences in maximal tested concentrations (2, 8 and 66 μM, respectively), and making meaningful comparison of these data difficult (the distribution of AUC estimates for crtizotinib and other drugs can be visualized with the QAPC portal, *Summary* tab). To solve this problem, we applied adjusted AUC, our new metric that takes into account the differences in the range of tested drug concentrations. The adjusted AUC uses sigmoid curve parameters estimated with a standard logistic regression (IC_50_ or EC_50_ models for adjusted AUC_IC50_ and adjusted AUC_EC50_, respectively), however, it is calculated only for the range of concentrations that is shared by the dose-response curves being compared (Figure 1B, green box). In contrast to IC_50_ capping, no data is discarded (non-overlapping high drug concentration data points are used for the sigmoid curve modeling).

To evaluate the performance of adjusted AUC_IC50_ and adjusted AUC_EC50_, and compare it to the traditional drug sensitivity metrics (IC_50_, EC_50_, and unadjusted AUC_IC50_and AUC_EC50_), we correlated drug sensitivity data obtained from CCLE, GDSC and CTRP (Figure 1C). There is a significant overlap in the drugs and cell lines analyzed by CCLE, GDSC and CTRP (Supplementary Figure S1, Supplementary Tables S3 and S4), permitting such analysis. Specifically, twelve drugs and 264 cell lines are represented in all 3 databases and pairwise intersection is even larger (Supplementary Figure S1).

First, we compared combined data for all compounds for the purpose of identifying the sensitivity metric that provides the best agreement between databases and, therefore, is the most reproducible quantitative assessment of the drug sensitivity for the pooled pharmacogenomic analysis. IC_50_, EC_50_ and unadjusted AUC drug sensitivity metrics produced mild to moderate agreement between the pharmacogenomic databases (Figure 1C, Supplementary Table S5, Supplementary Figures S2-S4, QAPC portal). When adjusted for the range of drug concentrations tested, CCLE drug sensitivity data agreed very well with that of CTRP (Pearson correlation, *r*, for adjusted AUC_IC50_ = 0.82), and moderately well with the pharmacologic data from GDSC (*r* for adjusted AUC_IC50_ = 0.69). An improvement in the agreement between drug sensitivities measured with adjusted AUC is particularly noticeable when CTRP data is included, which is expected, because the CTRP tested highest maximal drug concentration among 3 studies (Supplementary Table S1) that skews the CTRP unadjusted AUC data towards the higher values. Both CCLE and CTRP projects were performed by the Broad Institute and used the same proliferation assay (CellTiterGlo), which likely contributed to the good reproducibility between these two studies. The correlation between CTRP and GDSC data was moderate (*r* for adjusted AUC_IC50_ = 0.65). Flexible curve modeling (EC_50_, AUC_EC50_) did not provide an additional advantage (Figure 1C).

**Figure 2:**
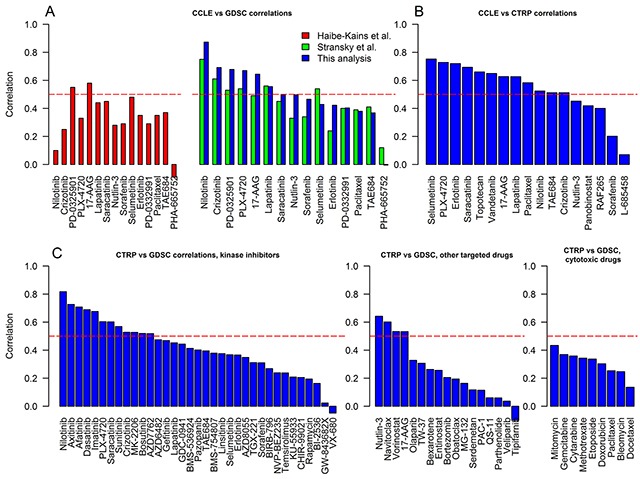
The correlations of cell line responses to individual drugs from CCLE, GDSC and CTRP **A.** CCLE and GDSC correlations. Pearson correlation (*r*) coefficients of adjusted AUC_IC50_ calculated by this study (blue bars) were matched to the similar analyses performed by Haibe-Kains et al. [5] (red bars, unadjusted AUC, Spearman correlation (ρ)), and Stransky et al. [6] (green bars, unadjusted AUC, *r*). **B.** CCLE and CTRP correlations (*r,* adjusted AUC_IC50_). **C.** CTRP and GDSC correlations (drugs are subdivided into kinase inhibitors, other targeted therapies, and cytotoxic chemotherapies; *r,* adjusted AUC_IC50_). Horizontal dashed line shows a threshold for moderate correlation (> 0.5).

The main purpose of pharmacogenomics is to associate molecular features with the sensitivity to a particular drug, therefore we studied whether the correlations hold when data is stratified by an individual compound (Figure 2, Supplementary Table S5, QAPC Portal). For most drugs the highest correlation was achieved with the adjusted AUC_IC50_ (Supplementary Table S5, QAPC Portal), supporting the superiority of our drug sensitivity metric. The *r* value for the adjusted AUC_IC50_ was significantly higher than *any* other unadjusted drug sensitivity metric for lapatinib (Supplementary Figure S5) and selumetinib in the CCLE-CTRP comparison, and for afatinib, axitinib, gefitinib, and nilotinib in the CTRP-GDSC comparison (higher *r* with non-overlapping 95% confidence intervals obtained with random permutations, Supplementary Table S5). Because adjusted AUC_IC50_ outperformed other drug sensitivity metrics, we chose it to assess the agreement between the databases and to compare our analysis with previously published analyses.

Adjusted AUC_IC50_ resulted in an improved correlation of the drug sensitivity data in CCLE and GDSC, when compared to the similar analyses (that used unadjusted AUC) performed by Haibe-Kains et al. [5] and Stransky et al. [6] (Kruskal-Wallis, p = 0.02; paired Mann-Whitney-Wilcoxon (this analysis vs Stransky et al. analysis), p = 0.05). The correlation has improved by > 0.1 for 6 out of 15 drugs: nilotinib, PD-0325901, PLX-4720, nutlin-3, sorafenib, and erlotinib, (Figure 2A, Supplementary Table S5). As expected, CCLE and CTRP drug sensitivities agreed well (Figure 2B), with 12 out of 17 drugs demonstrating at least a moderate degree of correlation (*r* > 0.5). Sixty one drugs were analyzed by both GDSC and CTRP allowing us to subdivide compounds based on the primary mechanism of action. The drug responses of kinase inhibitors showed the best agreement with 12 out 35 (34%) drugs with *r* > 0.5. Only 4 out of 17 (24%) other targeted therapies demonstrated moderate correlation. Surprisingly, drug sensitivity data for cytotoxic drugs showed minimal or poor correlation. (*r* < 0.5).

Close examination of the scatterplots and raw dose response curves for the targeted therapies with the help of the QAPC portal demonstrated that most drugs with very low *r* show no or low activity in the proliferation assay, explaining the lack of correlation. The variability of drug responses for inactive compounds is likely due to the intrinsic noise of the high-throughput screening and has no biologic meaning. This is true for PHA−665752 (Supplementary Figure S6) and sorafenib (CCLE and GDSC comparison), L−685458 and sorafenib (CCLE and CTRP comparison), bexarotene, BIRB-796, CHIR−99021, erlotinib, KU−55933, olaparib, PAC−1, parthenolide, QS−11, serdemetan, sorafenib, TGX−221, veliparib, and VX−680 (CTRP and GDSC comparison). The scatterplots for the individual drugs are not included in this paper due to the space limitations but can be examined using the QAPC Portal (*Correlation Plots* tab) or downloaded together with the with R bioinformatics pipeline (“fig” folder, Supplementary Figures 2-4; http://tanlab.ucdenver.edu/QAPC/Downloads/Drug_Sensitvity_Metrics.zip).

To improve the accessibility of the drug sensitivity data, we re-calculated 6 drug sensitivity metrics for all drugs and cell lines in CCLE, GDSC and CTRP (including adjusted AUC for the drugs analyzed by more than one database) and have made this data available for downloading through the QAPC portal. Alternatively, AUC can be calculated for the specified range of drug concentrations using functions from the recently published PharmacoGx package in *R* [10] (at this time, the PharmacoGx analysis is limited to the data from CCLE and GDSC databases) or by using the code from our downloadable bioinformatics pipeline.

In conclusion, we developed a new drug sensitivity metric, AUC adjusted for the range of tested concentrations, which allows reconciliation of publicly available heterogeneous pharmacologic data enabling pooled analysis for drug/gene associations. Using this methodology we have achieved the best correlation to date between overlapping data in CCLE, GDSC and CTRP databases, and we demonstrated that there is a moderate-to-good agreement between the drug sensitivity data for many targeted therapies, particularly for kinase inhibitors with activity in an *in vitro* proliferation assay. We predict that the adjusted AUC can be used to match *in vitro* drug responses generated by the high-throughput testing of patient-derived primary tumor cells with an existing pool of data to rapidly identify outlier drug sensitivities with potential therapeutic implications.

While our method accounts for several differences in the data analysis and experimental design, we acknowledge, that the variability introduced by the intrinsic features of each study (proliferation assay, cell line passage, growth media, plating density and others) outlined in the supplementary data for Haibe-Kains et al. [5] is unlikely to be alleviated with bioinformatics analysis alone.

## MATERIALS AND METHODS

### Data

Raw dose-response data was downloaded from CCLE portal (http://www.broadinstitute.org/ccle) [1], GDSC website (http://www.cancerrxgene.org/downloads/) [2], and The National Cancer Institute's CTD2 Network (CTRP2, https://ctd2.nci.nih.gov/dataPortal/) [3].

### Calculations of drug sensitivity metrics

Log-logistic regression was used to model sigmoid dose response curve using the following formula:

$$y=A_{\max}+\left(\frac{A_{\min}-A_{\max}}{1+{\left(\frac{x}{EC_{50}}\right)}^{Hill}}\right)$$ (1)

where *A_min_* and *A_max_* are lower and upper asymptotes of the sigmoid curve (no response and the maximal response to the drug, respectively), EC_50_ is drug concentration causing the effect equal to the 50% of the *A_max_*, and *Hill* is a Hill slope of the dose response curve. R package *drc* [11], www.bioassay.dk, was used for modeling. 4-point regression analysis was performed first, and minimal and maximal asymptotes were allowed within the following ranges: *R_min_* ≤ *A_min_* ≤ 0 and min(0, *R_min_*) ≤ *A_max_* ≤ max (100, *R_max_*), respectively. *R_min_* and *R_max_* are minimal and maximal measured drug responses, respectively. The coordinate of the upper bend point of the dose response curve was calculated using the formula [12]:

$$x_{\text{bend}}=\left(\frac{A_{\min}-A_{\max}}{1+4.6805}\right)+A_{\max}$$ (2)

If two or more data points were present with the drug concentration x > x_bend_, the curve was assumed to be complete and the upper asymptote estimated accurately [13]. Otherwise, regression analysis was repeated with the *A_max_ =* 100 (for the lack of a better upper asymptote estimate for incomplete curves). *A_max_ =* 100 was also set for very low amplitude curves (*A_max_* - *A_min_* < 30) to avoid the noise being misinterpreted as a true drug response. To estimate IC_50_, *A_min_* and *A_max_* were set to 0 and 100, respectively (by the definition of IC_50_). The parameters of the sigmoid curve were used for AUC calculations.

AUC was estimated in between minimal (x_min_) and maximal (x_max_) tested concentrations (nM) for the curve. For the purpose of comparing two dose response curves, an adjusted AUC was calculated for the range of concentrations shared by both curves (Figure 2B, green box). AUC was calculated using the following formula:

$$\text{AUC}=\frac{\sum_{x=x_{\min}}^{x_{\max}}\left(f\left(x\right)-A_{\min}\right)}{\sum_{x=x_{\min}}^{x_{\max}}\left(\max\left(A_{\max},100\right)-A_{\min}\right)}$$ (3)

where *f(x)* = formula (1)

To perform an unbiased comparison of EC_50_ and IC_50_, the estimated values that exceed maximal tested concentration were set to infinity acknowledging the fact, that EC_50_ and IC_50_ cannot be accurately estimated from incomplete dose response curves.

### Comparisons of drug sensitivity metrics

Pearson and Spearman correlations were used to compare drug sensitivity data between studies. Pearson correlation for pooled EC_50_ and IC_50_ data was calculated for a subset of finite values. p-values were computed by performing random permutations and bootstrapping using published algorithm [6]. P-values and confidence intervals calculated using random permutations are cited in the text.

### Implementation and source code

All data manipulations were performed in R (https://www.r-project.org/). To ensure reproducibility, all figures and tables of this manuscript were generated programmatically from the raw data. R scripts and data can be downloaded from: http://tanlab.ucdenver.edu/QAPC/Downloads/Drug_Sensitvity_Metrics.zip

### QAPC portal

All aspects of this analysis can be explored interactively using companion portal the Quantitative Analysis of Pharmacogenomics in Cancer (QAPC, http://tanlab.ucdenver.edu/QAPC/).

QAPC features include:

1 Interactive graphic interface to access drug sensitivity data for 585 drugs/drug combinations and 1201 cell lines.
2 Choice of 6 drug sensitivity metrics:
  EC_50_ – half maximal effective concentration
  IC_50_ – half maximal inhibitory concentration
  AUC EC_50_ – area under the dose response curve calculated from EC_50_ model
  AUC IC_50_ – area under the dose response curve calculated from IC_50_ model
  Adjusted AUC EC_50_ – area under the dose response curve calculated from EC_50_ model adjusted for the range of tested drug concentrations
  Adjusted AUC IC_50_ – area under the dose response curve calculated from IC_50_ model adjusted for the range of tested drug concentrations
3 Option to reconcile data from up to three databases using adjusted AUC EC_50_ or adjusted AUC IC_50_. Adjusted AUC for the cell line/drug pairs analyzed by more than one study are averaged. Adjusted AUC are calculated even for the cell lines analyzed by one database only, if the drug is represented in more than one database, thus allowing a fair comparison of drug sensitivities across studies and enabling high-power pharmacogenomics analysis.
4 Functionality to visualize dose-response curves for each drug/cell line combination to evaluate the quality of the raw data and the accuracy of the dose response model.
5 Graphic representation of the drug sensitivity data agreement between CCLE, GDSC and CTRP databases with 2D and 3D scatterplots.
6 Functionality to download raw data, EC_50_ and IC_50_ regression model parameters, adjusted AUC calculations, and correlation statistics for the drug.

Detailed description of the QAPC controls and interface can be accessed through the *Help* tab of the online portal.

## SUPPLEMENTARY FIGURE AND TABLES










