# Supplementary material for: Integrating heterogeneous drug sensitivity data from cancer pharmacogenomic studies
Source: Oncotarget. 2016 Jun 14;7(32):51619–25. doi: 10.18632/oncotarget.10010 (PMC5239501; doi:10.18632/oncotarget.10010)
Supplement: Supplementary file 1 [file oncotarget-07-51619-s001.pdf]

# Integrating heterogeneous drug sensitivity data from cancer pharmacogenomic studies

## SUPPLEMENTARY FIGURE AND TABLES

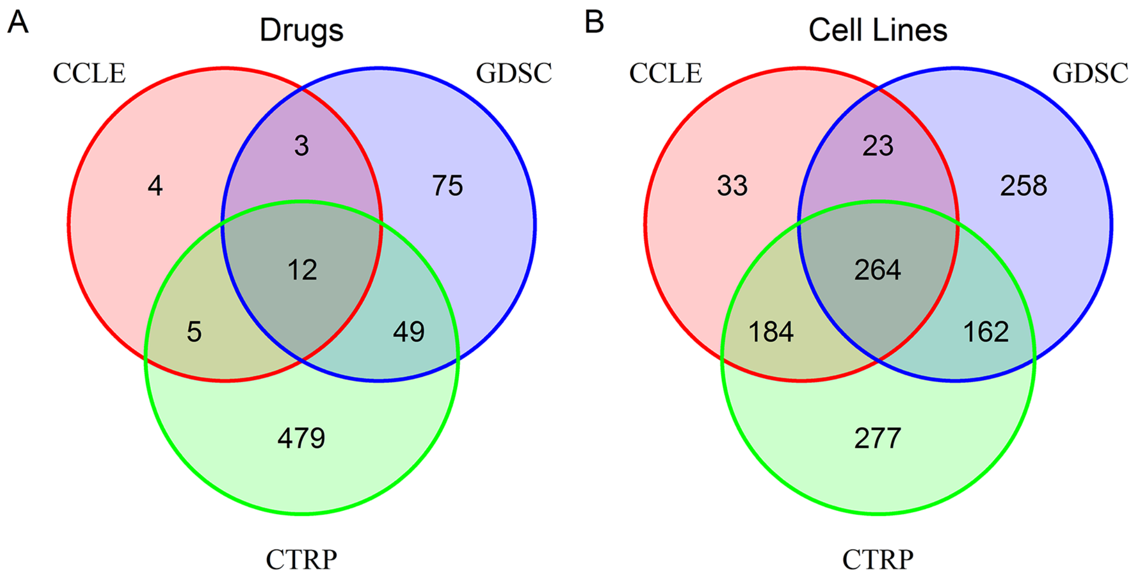


**Supplementary Figure S1: The intersection between CCLE, GDSC, and CTRP databases in terms of drugs A. and cell lines B.**

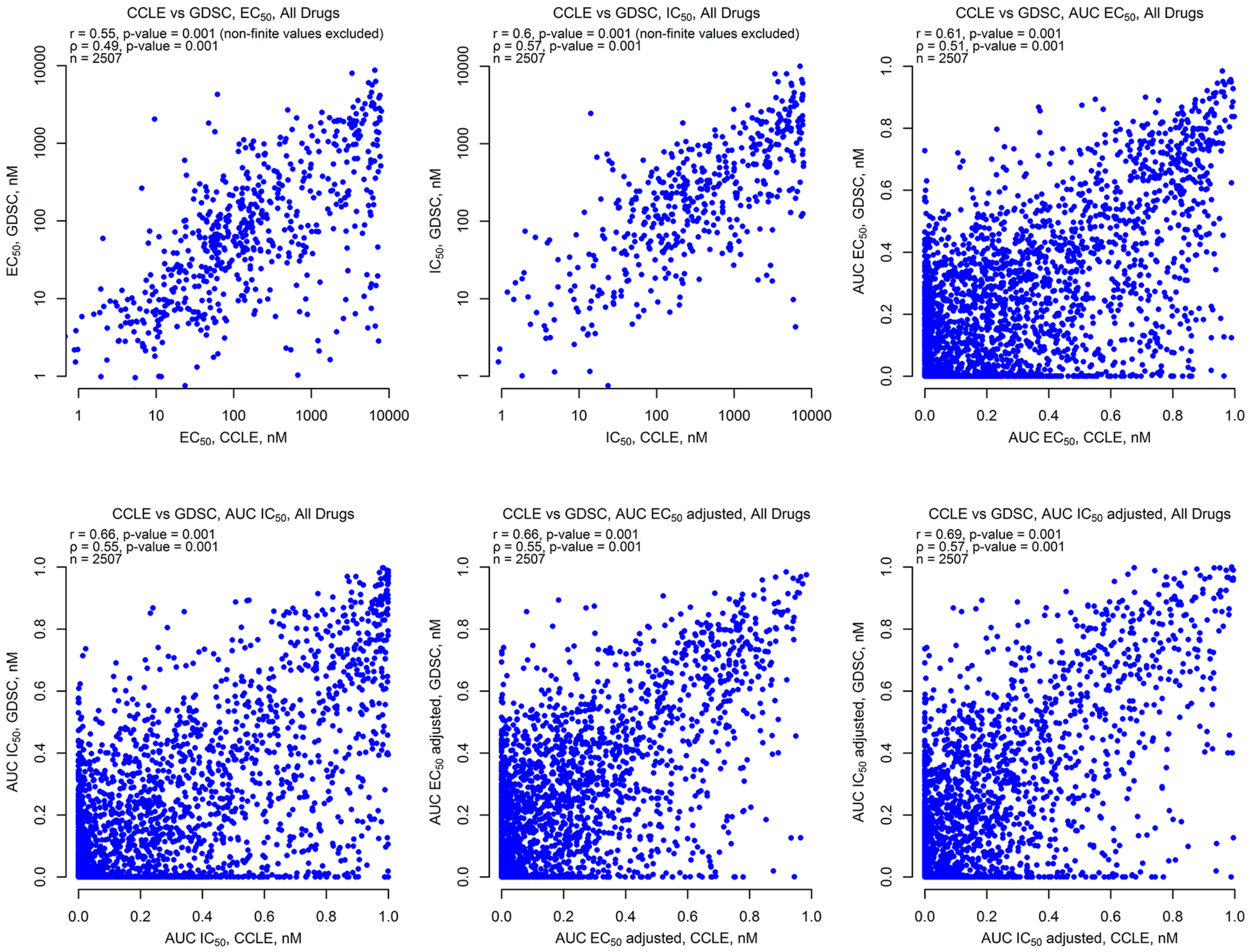


**Supplementary Figure S2: The agreement of drug sensitivities from CCLE and GDSC databases.** Scatterplots for pooled data analyzed with 6 drug sensitivity metrics are shown. Infinite $EC_{50}$ and $IC_{50}$ values are not visualized on the plots. Pearson (*r*) and Spearman (ρ) correlations, p-values, and the number of data points are shown for each plot. Pearson correlation was calculated for a subset of $EC_{50}$ and $IC_{50}$ with finite values (estimated to be within the range of tested concentrations).

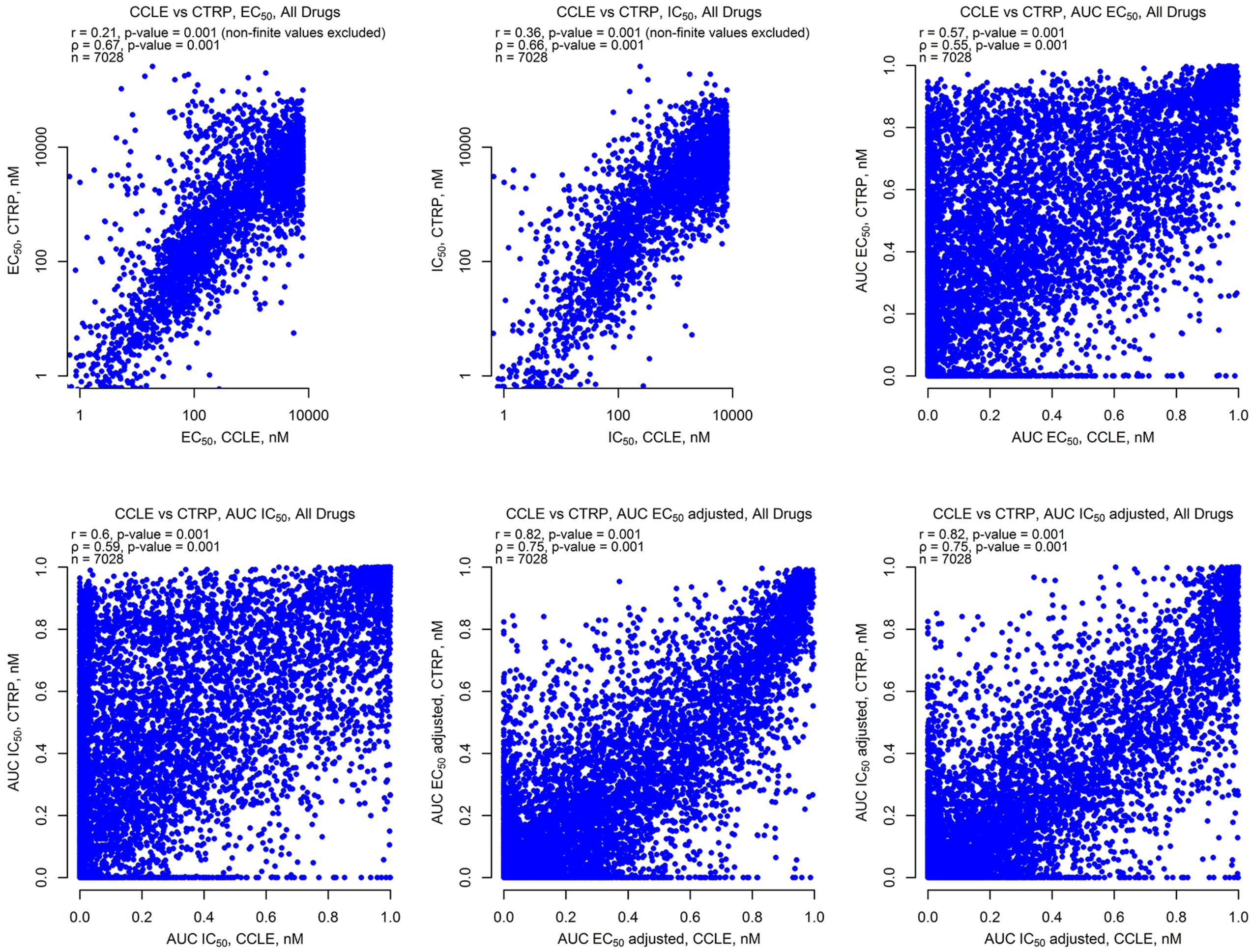


**Supplementary Figure S3: The agreement of drug sensitivities from CCLE and CTRP databases.** Scatterplots for pooled data analyzed with 6 drug sensitivity metrics are shown. Infinite $EC_{50}$ and $IC_{50}$ values are not visualized on the plots. Pearson (*r*) and Spearman (ρ) correlations, p-values, and the number of data points are shown for each plot. Pearson correlation was calculated for a subset of $EC_{50}$ and $IC_{50}$ with finite values (estimated to be within the range of tested concentrations).

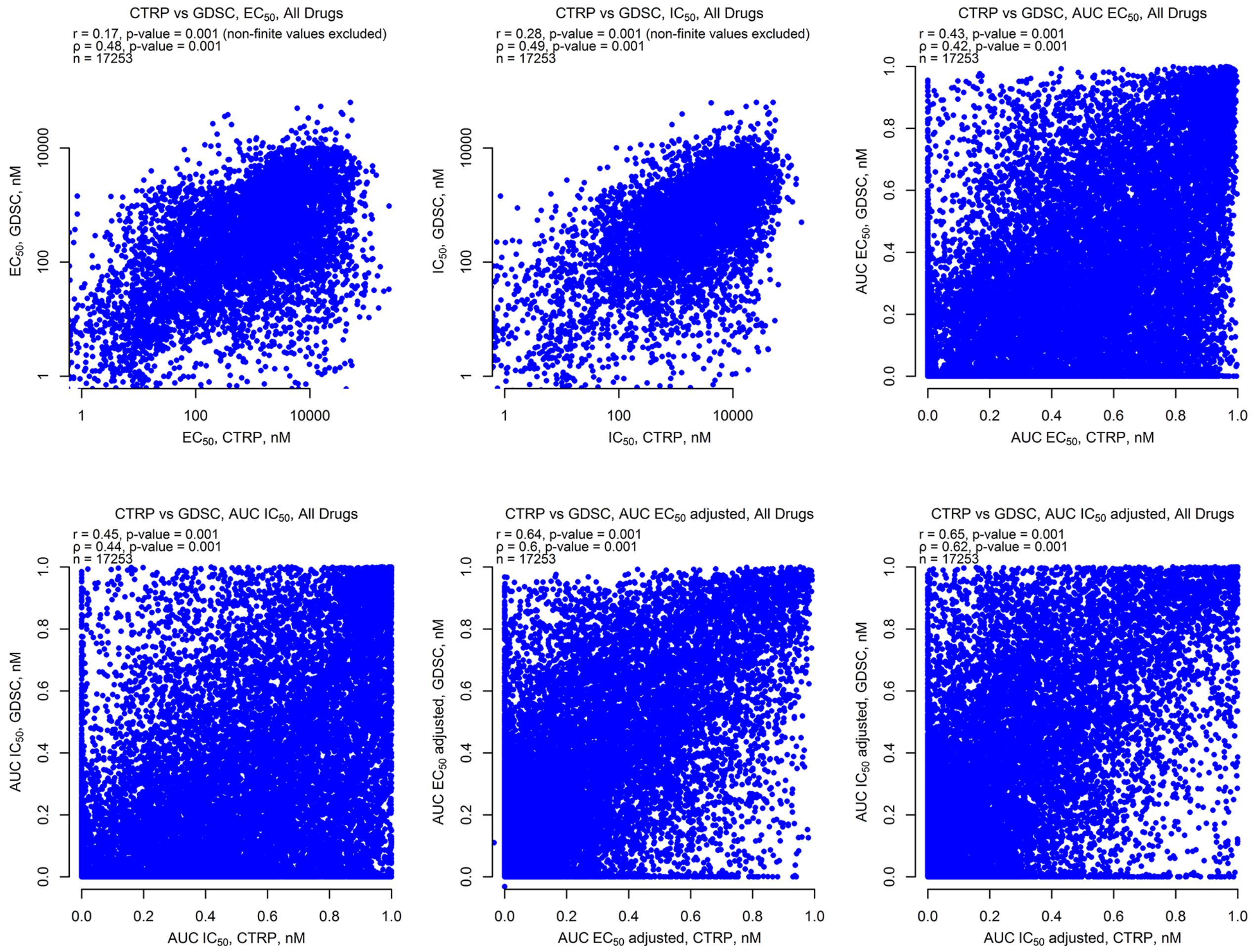


**Supplementary Figure S4: The agreement of drug sensitivities from CTRP and GDSC databases.** Scatterplots for pooled data analyzed with 6 drug sensitivity metrics are shown. Infinite $EC_{50}$ and $IC_{50}$ values are not visualized on the plots. Pearson (*r*) and Spearman (ρ) correlations, p-values, and the number of data points are shown for each plot. Pearson correlation was calculated for a subset of $EC_{50}$ and $IC_{50}$ with finite values (estimated to be within the range of tested concentrations).

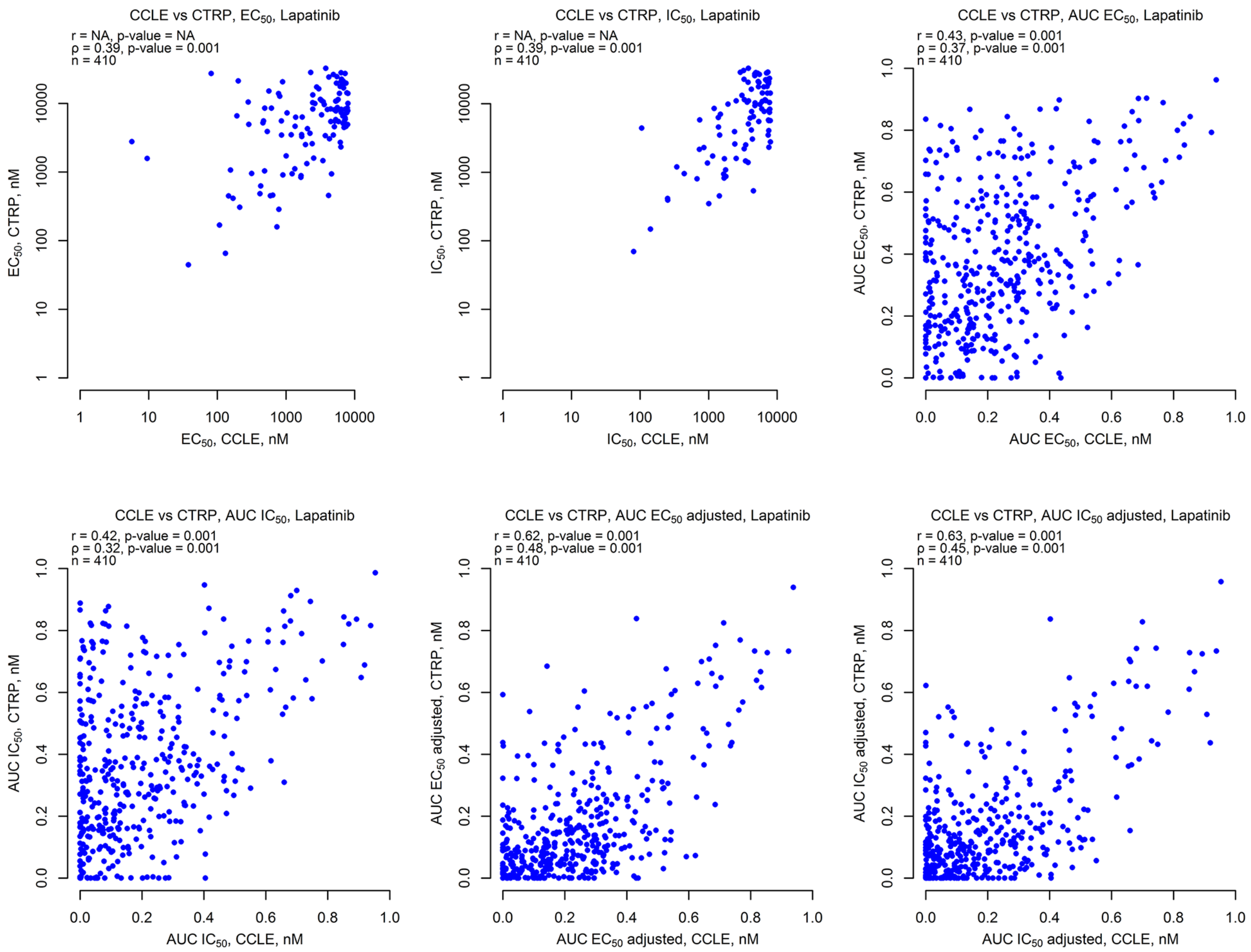


**Supplementary Figure S5: Adjusted for the range of tested concentrations AUC outperforms other drug sensitivity metrics.** Scatterplots for the drug sensitivity data for lapatinib from CCLE and CTRP databases analyzed with 6 drug sensitivity metrics are shown. Infinite $EC_{50}$ and $IC_{50}$ values are not visualized on the plots. Pearson (*r*) and Spearman (ρ) correlations, p-values, and the number of data points are shown for each plot.

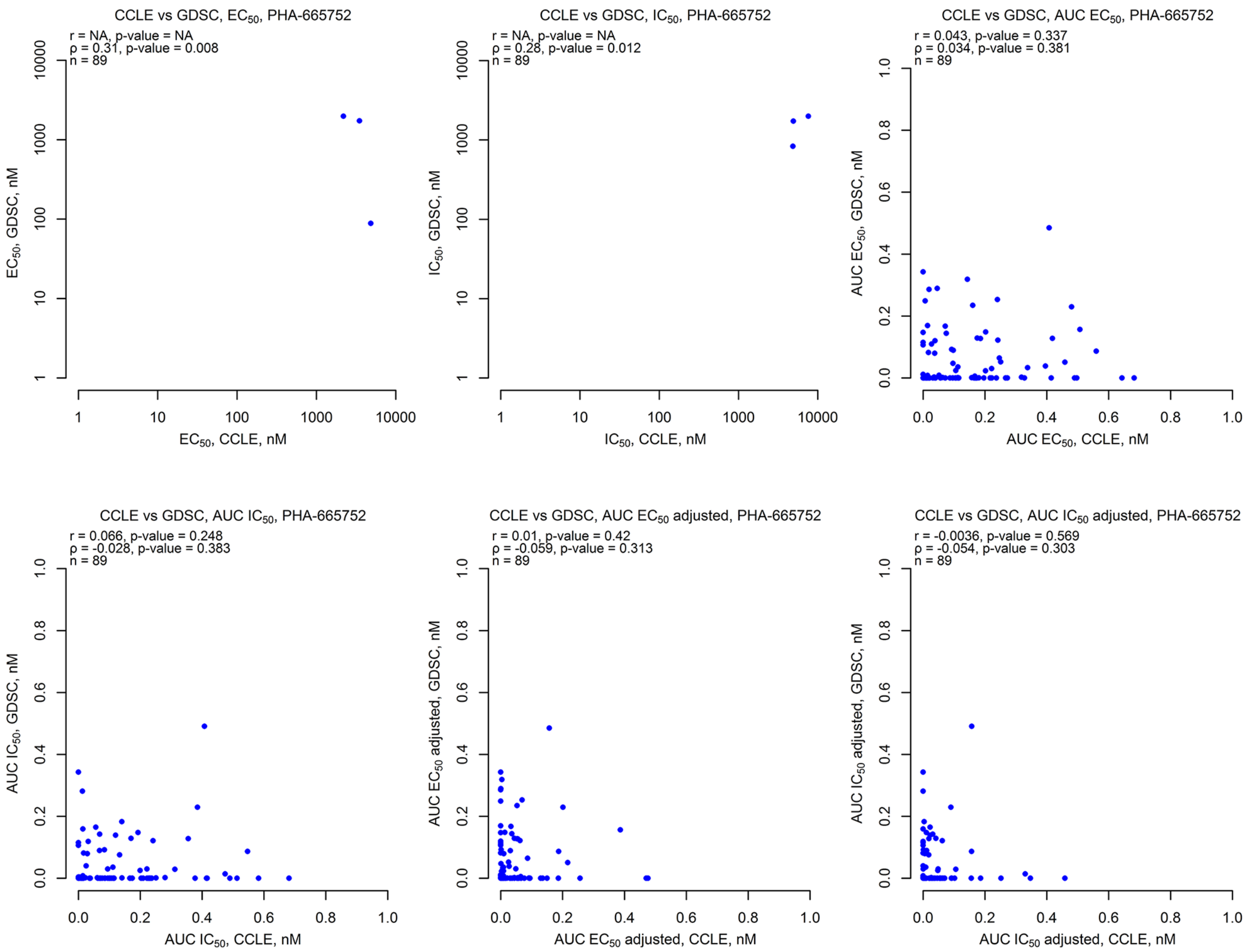


**Supplementary Figure S6: Drug responses for PHA-665752, that has no/limited activity in an *in vitro* proliferation assay, correlate poorly in CCLE and GDSC databases.** Infinite EC$_{50}$ and IC$_{50}$ values are not visualized on the plots. Pearson (*r*) and Spearman (ρ) correlations, p-values, and the number of data points are shown for each plot.

**Supplementary Table S1: Maximal tested concentrations (nM) for drugs in CCLE, GDSC and CTRP databases.**

**See Supplementary File 1**

**Supplementary Table S2: Percentages of $IC_{50}$ values capped to the maximal tested concentration in the Stransky et al. [6] analysis**

| Drug | % of capped $IC_{50}$ values | |
|---|---|---|
| | CCLE | GDSC |
| 17-AAG | 11 | 27 |
| Saracatinib | 89 | 87 |
| Selumetinib | 76 | 76 |
| Erlotinib | 93 | 95 |
| Lapatinib | 90 | 95 |
| Nilotinib | 95 | 98 |
| Nutlin-3 | 94 | 84 |
| Paclitaxel | 22 | 37 |
| PD0325901 | 79 | 72 |
| PD0332991 | 92 | 71 |
| Crizotinib | 95 | 94 |
| PHA665752 | 98 | 99 |
| PLX4720 | 0 | 89 |
| Sorafenib | 93 | 90 |
| TAE684 | 75 | 51 |

$IC_{50}$ values were capped to the maximal concentration tested by GDSC, which varies by drug (Supplementary Table S1).

**Supplementary Table S3: Drugs tested by CCLE, GDSC and CTRP.** Each row corresponds to the one drug. Empty cells indicate that the drug was not used in the study identified in a column name. The fourth column contains consensus drug names used in this analysis.

**See Supplementary File 2**

**Supplementary Table S4: Cell lines tested by CCLE, GDSC and CTRP.** Each row corresponds to the one cell line. Empty cells indicate that the cell line was not used in the study identified in a column name. The fourth column lists consensus cell line names used in this analysis.

**See Supplementary File 3**

**Supplementary Table S5: Correlation statistics for comparisons of drug sensitivity estimates in CCLE, GDSC and CTRP databases.** Columns are labeled as follows: database1 and database2 – source databases; drug – name of the drug ("All" for pooled data from all drugs); drug_sensitivity_metric – one of the six drug sensitivity metrics: EC50 - half maximal effective concentration; IC50 - half maximal inhibitory concentration; AUC_EC50 - area under the curve calculated from the $EC_{50}$ model; AUC_IC50 - area under the curve calculated from the $IC_{50}$ model; AUC_EC50_adj - area under the curve calculated from $EC_{50}$ model adjusted for the range of tested drug concentrations; AUC_IC50_adj - area under the curve calculated from $IC_{50}$ model adjusted for the range of tested drug concentrations; correlation_type - Pearson or Spearman; correlation – correlation coefficient; p.permutations - p-value calculated by random permutations; p.bootstrap - p-value calculated by bootstrapping; confidence.interval - 95% confidence interval calculated by random permutations; data_points - number of data points in the comparison.

**See Supplementary File 4**
