# Supplementary material for: Integrating heterogeneous drug sensitivity data from cancer pharmacogenomic studies
Source: Oncotarget. 2016 Jun 14;7(32):51619–25. doi: 10.18632/oncotarget.10010 (PMC5239501; doi:10.18632/oncotarget.10010)
Supplement: Supplementary file 2 [file oncotarget-07-51619-s002.docx]

| **Drug** | **CCLE** | **GDSC** | **CTRP** |
| --- | --- | --- | --- |
| 17-AAG | 8000 | 1000 | 8300, 17000 |
| Afatinib |  | 500 | 260, 17000, 66000 |
| Axitinib |  | 2000 | 8300, 17000, 33000, 66000 |
| AZD6482 |  | 5000, 5120 | 33000, 66000 |
| AZD7762 |  | 2000 | 33000, 66000 |
| AZD8055 |  | 2000 | 17000, 33000, 66000 |
| Bexarotene |  | 8000 | 8300, 17000, 33000, 66000 |
| BI-2536 |  | 256 | 33000 |
| BIRB-796 |  | 10000 | 260, 17000, 33000, 66000 |
| Bleomycin |  | 64000 | 17000, 33000, 66000 |
| BMS-536924 |  | 5120 | 580, 4600, 18000, 37000 |
| BMS-754807 |  | 2560 | 4200, 17000, 33000 |
| Bortezomib |  | 20 | 66, 130 |
| Bosutinib |  | 2000 | 17000, 33000 |
| CHIR-99021 |  | 2560 | 40000, 80000 |
| Crizotinib | 8000 | 2000 | 33000, 66000 |
| Cytarabine |  | 2000 | 17000, 33000, 66000 |
| Dasatinib |  | 1280 | 33000, 66000 |
| Docetaxel |  | 12.5 | 33000, 66000 |
| Doxorubicin |  | 1024 | 33000, 66000 |
| Entinostat |  | 5000 | 33000, 66000 |
| Erlotinib | 8000 | 2000 | 33000, 66000 |
| Etoposide |  | 16000 | 28000 |
| GDC-0941 |  | 2000, 4000 | 1000, 17000, 33000, 66000 |
| Gefitinib |  | 500 | 8300, 17000, 33000 |
| Gemcitabine |  | 1024 | 8300, 33000 |
| GW-843682X |  | 256 | 8300, 17000, 33000 |
| Imatinib |  | 2000 | 33000, 66000 |
| KU-55933 |  | 10000 | 33000, 66000 |
| L-685458 | 8000 |  | 140, 4600, 9200, 18000, 37000 |
| Lapatinib | 8000 | 2000 | 260, 17000, 33000 |
| Methotrexate |  | 200 | 260, 33000, 66000 |
| MG-132 |  | 1000 | 20000 |
| Mitomycin |  | 16000 | 3100, 25000, 50000 |
| MK-2206 |  | 2000, 4000 | 8300, 17000, 33000 |
| Navitoclax |  | 2000 | 1000, 8300, 17000 |
| Nilotinib | 8000 | 2000 | 17000, 33000, 66000 |
| Nutlin-3 | 8000 | 4000, 8000 | 150000 |
| NVP-BEZ235 |  | 250, 2000 | 8300, 17000 |
| Obatoclax |  | 16000 | 260, 17000, 33000, 66000 |
| Olaparib |  | 5000 | 580, 74000, 150000 |
| Linsitinib |  | 2560 | 2100, 8300, 17000, 33000 |
| PAC-1 |  | 2560 | 8300, 17000, 33000 |
| Paclitaxel | 8000 | 102.4 | 4200 |
| Panobinostat | 8000 |  | 17000, 33000, 66000 |
| Parthenolide |  | 5000 | 260, 17000, 33000, 66000 |
| Pazopanib |  | 8000 | 17000, 33000, 66000 |
| PD-0325901 | 8000 | 250, 2000 |  |
| PD-0332991 | 8000 | 2000, 4000 |  |
| PHA-665752 | 8000 | 2000 |  |
| PLX-4720 | 2530, 8000 | 10000 | 8300, 17000, 33000 |
| QS-11 |  | 10240 | 8300, 17000, 33000 |
| RAF265 | 2530, 8000 |  | 260, 17000, 33000 |
| Rapamycin |  | 100 | 8300, 17000, 33000 |
| Saracatinib | 8000 | 2000 | 33000, 66000 |
| Selumetinib | 8000 | 2000, 4000 | 66000, 130000, 270000 |
| Serdemetan |  | 10000 | 8300, 17000, 33000 |
| Sorafenib | 8000 | 4000 | 17000, 33000 |
| Sunitinib |  | 8000 | 8300, 66000 |
| TAE684 | 8000 | 2000 | 17000, 33000 |
| Temsirolimus |  | 200 | 8300, 17000, 33000, 66000 |
| TGX-221 |  | 10000 | 17000, 33000, 66000 |
| Tipifarnib |  | 16000 | 33000 |
| Topotecan | 8000 |  | 260, 33000, 66000 |
| TW-37 |  | 5000 | 520, 2100, 4200, 8300, 17000, 33000 |
| Vandetanib | 8000 |  | 260, 17000, 33000 |
| Veliparib |  | 5000 | 130, 8300, 17000, 33000 |
| Vorinostat |  | 10000 | 17000, 33000 |
| VX-680 |  | 2000 | 66000 |
